# Yinchen Linggui Zhugan Decoction Ameliorates Nonalcoholic Fatty Liver Disease in Rats by Regulating the Nrf2/ARE Signaling Pathway

**DOI:** 10.1155/2017/6178358

**Published:** 2017-08-28

**Authors:** Yi Guo, Jun-xiang Li, Yun-liang Wang, Tang-you Mao, Chen Chen, Tian-hong Xie, Ya-fei Han, Xiang Tan, Hai-xiao Han

**Affiliations:** ^1^Beijing University of Chinese Medicine, No. 11, North Third Ring East Road, Beijing 100029, China; ^2^Gastroenterology Department, Dongfang Hospital, Beijing University of Chinese Medicine, No. 6, 1st Section, Fangxingyuan, Fangzhuang, Beijing 100078, China

## Abstract

Yinchen Linggui Zhugan Decoction (YCLGZGD) is the combination of Linggui Zhugan (LGZGD) and Yinchenhao (YCHD) decoctions, two famous traditional Chinese medicine prescriptions. In previous studies, we found that Yinchen Linggui Zhugan Decoction (YCLGZGD) could regulate lipid metabolism disorder and attenuate inflammation in pathological process of nonalcoholic fatty liver disease (NAFLD). However, the exact underlying mechanism remains unknown. The aim of this study was to explore the effect of Yinchen Linggui Zhugan Decoction on experimental NAFLD and its mechanism in rats with high-fat diet (HFD) which was established by 8-week administration of HFD. YCLGZGD, LGZGD, and YCHD were administered daily for 4 weeks, after which the rats were euthanized. The level of blood lipid, liver enzymes, H&E, and Oil Red O staining were determined to evaluate NAFLD severity. Western blotting and real-time polymerase chain reaction were, respectively, used to determine hepatic protein and gene expression of Keap1, Nrf2, NQO1, and HO-1. Oral YCLGZGD ameliorated HFD-induced NAFLD. Furthermore, YCLGZGD increased the protein and gene expression of Nrf2, NQO1, and HO-1 without changing Keap1. Overall, these results suggest that YCLGZGD ameliorates HFD-induced NAFLD in rats by upregulating the Nrf2/ARE signaling pathway.

## 1. Introduction

Nonalcoholic fatty liver disease (NAFLD) is a type of liver disease that includes simple hepatic steatosis, nonalcoholic steatohepatitis (NASH), and irreversible cirrhosis [1]. The prevalence of NAFLD has rapidly increased in parallel to dramatic rise in obesity, diabetes [2, 3], hypertension [4, 5], and dyslipidemia [6]. Currently, NAFLD is regarded as the liver manifestation of metabolic syndrome. The pathogenesis of NAFLD is not fully understood thus far and the reported therapeutic trails are still under investigation [7, 8]. Some researchers agree with the “2-hit” hypothesis for the NAFLD pathogenesis. Briefly, the “first hit” involves hepatic triglyceride accumulation or steatosis. The “second hit” represents the relationship between inflammatory cytokines and oxidative stress. The Nrf2/antioxidant response element (ARE) signaling pathway plays an important role in oxidative stress and can induce the expression of antioxidative genes to protect hepatocytes from apoptosis.

NAFLD management strategies often include lifestyle modifications and pharmaceutical interventions [9–12]. However, compliance with the long-term lifestyle modification is poor and most medicines have adverse effects, which limit their usage [13]. Thus, it is necessary to develop novel strategies with fewer side effects and high therapeutic efficiency.

Chinese herbal medicine (CHM) has been traditionally used in China and other Asian countries for thousands of years and its use is now spreading worldwide. A unique and basic feature of CHM is the use of a formula containing several herbs (mixed as a cocktail) to ameliorate various abnormalities related to a certain disease. Herbal extracts contain multiple natural compounds that can target various pathological pathways underlying a disease, providing therapeutic effects via a range of mechanisms. Linggui Zhugan (LGZGD) and Yinchenhao (YCHD) decoctions are two well-known traditional CHM, from Treatise on Febrile Diseases, and consist of Fuling* (Poria cocos)*, Guizhi* (cassia twig)*, Baizhu* (Atractylodes macrocephala *Koidz), Gancao* (licorice)*, Yinchen* (herba artemisiae scopariae)*, Zhizi* (Gardenia)*, and Dahuang* (rhubarb)*. They are widely used to treat obesity and diabetes. As for chemical composition of YCHD and LGZGD, some previous researches were conducted to discuss effective components partly in two formulas by HPLC fingerprints. Catechins, anthraquinones, iridoids, crocetin, and chlorogenic acid can be responsible for curative effect of YCHD mainly [14], and LGZGD had therapeutic effect because of almost twenty compounds such as cinnamic acid, glycyrrhizic acid, and dehydrotumulosic acid [15].

Owing to synergistic effect, their combination is also used to treat NASH in clinics. In previous studies, we found that Yinchen Linggui Zhugan Decoction (YCLGZGD) has an anti-inflammatory effect [16, 17], which could regulate lipid metabolism disorder and attenuate inflammation in pathological process of NAFLD. Therefore, it is necessary to explore the difference in therapeutic effect of combination of compounds/herbs versus that of a single herb/compound and its possible mechanism.

## 2. Materials and Methods

### 2.1. Preparation of LGZGD, YCHD, YCLGZGD, and Sulforaphane (SFN)

LGZGD, YCHD, and YCLGZGD granules were provided by Pharmacy Department of Dongfang Hospital, Beijing University of Chinese Medicine (Beijing, China). All granules contain equal amounts of ingredients of the LGZGD decoction (Fuling 12 g, Guizhi 9 g, Baizhu 6 g, and Gancao 6 g) and YCHD decoction (Yinchen 18 g, Zhizi 9 g, and Dahuang 6 g). Yinchen Linggui Zhugan Decoction is a combination of these two decoctions. SFN was purchased from LKT Laboratories (St. Paul, Minnesota, USA).

### 2.2. Animals and Treatment

Male Sprague-Dawley (SD) rats (7-week-old) were supplied by SPF Biotechnology Co. Ltd. (Beijing, China). All experimental procedures were approved by the Animal Ethics Committee of Beijing University of Chinese Medicine (number 2015BZHYLL0201) and followed the Regulations for Laboratory Animal Management. SD rats were maintained on a 12 h light/dark cycle at 22 ± 2°C with ad libitum access to a standard chow diet (*n* = 10) or high-fat diet (HFD, 34% fat, 19% protein, and 47% carbohydrate by energy composition) (*n* = 10) for 8 weeks to induce NAFLD. The granules and SFN were dissolved in 100 mL of distilled water and kept at 2–8°C until used. The rats received LGZGD, YCHD, and YCLGZGD (3.465 g/kg/day, 3.465 g/kg/day, and 6.93 g/kg/day, p.o., *n* = 10, resp.), and SFN (0.5 mg/kg/day, p.o., *n* = 10) after 8 weeks of HFD feeding. The rats in control group (fed chow; *n* = 10) received saline (10 mL/kg/day, p.o., *n* = 10). All groups were administered the drugs or saline for 4 weeks.

### 2.3. Determination of Metabolic Parameters: Liver Enzymes and Blood Lipid Levels in Rats

At the end of treatment, animals were anesthetized using 4% chloral hydrate after 12 h overnight fasting and blood samples were collected from the abdominal aorta. Fasting serum triglycerides (TGs), high-density lipoprotein cholesterol (HDL-C), and low-density lipoprotein cholesterol (LDL-C) were analyzed using enzyme-linked immunosorbent assay (ELISA) (Bio Sino, Beijing, China). Fasting serum alanine aminotransferase (ALT) and aspartate aminotransferase (AST) were also determined by ELISA, as previously described [18].

### 2.4. Histological Analyses

A fresh liver tissue sample was fixed with 10% formaldehyde solution. Paraffin-embedded sections were used for hematoxylin and eosin (H&E) staining (Ze-ping, Beijing, China). Frozen sections were stained with Oil Red O (ORO; Sigma Aldrich, St. Louis, Missouri, USA). Both staining methods were used to investigate architecture of the liver and hepatic lipid droplets. H&E and ORO-stained slides were visualized under a microscope (BX40, Olympus, Beijing, China), and images were captured with the attached digital camera using NIS Element SF 4.00.06 software (Beijing, China). For each group, liver samples from 3 to 5 rats were prepared and stained.

### 2.5. Western Blotting

To detect Keap1, Nrf2, NADPH quinone-oxidoreductase-1 (NQO1), and heme-oxygenase (HO-1) proteins, the liver tissue homogenates were extracted using ice-cold tissue lysis buffer. Protein concentration was determined using a BCA protein assay kit. Samples were separated by 10% SDS-PAGE and transferred onto polyvinylidene difluoride membranes. The membranes were immunoblotted with primary antibodies for* Keap1 *(1 : 1000)*, Nrf2 *(1 : 1000)*, NQO1 *(1 : 1000)*, HO-1 *(1 : 1000) (Abcam, USA), and*β-actin *(ZSGB-BIO, Beijing, China). Peroxidase-conjugated secondary antibodies and an ECL detection system were used according to routinely used methods as previously described [19]. The intensities of the protein bands were analyzed using Gel-Pro 3.2 software. *β*-Actin protein was used as the internal control to normalize the protein loading.

### 2.6. Real-Time Polymerase Chain Reaction for Keap1, Nrf2, NQO1, and HO-1 mRNA Expression

As previously described [20], reverse transcription was performed with 1 *μ*g of total RNA per 12 *μ*l reaction using a standard cDNA synthesis kit (Takara, Japan). The real-time PCR primer sequences for target genes were as follows: Keap1, forward 5′-TAACCGGCTTAACTCGGCAG-3′ and reverse 5′-GGAGGCTACGAAAGTCCAGG-3′; Nrf2, forward 5′-AGCAGGCTGAGACTACCACT-3′ and reverse 5′-TCCAGTGAGGGGATCGATGA-3′; NQO1, forward 5′-GATTGTATTGGCCCACGCAG-3′ and reverse 5′-GATTCGACCACCTCCCATCC-3′; HO, forward 5′-GGGTCCTCACACTCAGTTTC-3′ and reverse 5′-CCAGGCATCTCCTTCCATTC-3′; *β*-actin, forward 5′-CCCATCTATGAGGGTTACG-3′ and reverse 5′-TTTAATGTCACGCACGATTTC-3′ (CW-bio, Beijing, China). The PCR conditions were as follows: an initial activation step at 95°C for 5 min, 45 cycles of amplification, and a final melting curve (55–95°C). For comparison, the cDNA concentrations were normalized to *β*-actin PCR products. The data were analyzed by using the 2^−ΔΔCt^ method.

### 2.7. Data Analysis

All data are expressed as the mean ± SD, unless otherwise indicated. SPSS v20.0 (IBM Corp, Armonk, NY, USA) was used for statistical analyses. Data were analyzed by one-way ANOVA, followed by Student's *t*-tests. Differences were considered to be statistically significant at *P* < 0.05.

## 3. Results

### 3.1. Effect of LGZGD, YCHD, and YCLGZGD on Lipid Metabolism and Liver Enzymes in HFD-Fed Rats

**Figure 1:**
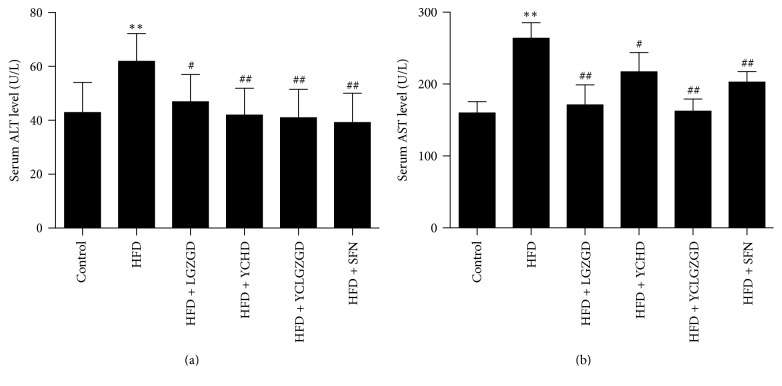
Effect of LGZGD, YCHD, and YCLGZGD on serum ALT and AST levels. (a) ALT levels in serum; (b) AST levels in serum. Data are means ± SD; *n* = 10/group; ^*∗∗*^*P* < 0.01 versus control; ^#^*P* < 0.05 and ^##^*P* < 0.01 versus HFD.

All animals tolerated the experimental procedures well, and no deaths occurred during the study. Serum ALT (62.50 ± 9.66 U/L) and AST (266.00 ± 19.30 U/L) concentrations in HFD-fed rats were significantly higher than those in chow-fed rats [ALT (43.50 ± 10.50 U/L) and AST (162.14 ± 13.50 U/L); *P* < 0.01]. Treatment with LGZGD [ALT (47.52 ± 11.10 U/L), AST (173.33 ± 25.60 U/L)], YCHD [ALT (42.55 ± 9.35 U/L), AST (219.40 ± 24.56 U/L)], YCLGZGD [ALT (41.55 ± 9.96 U/L), AST (164.56 ± 14.60 U/L)], SFN [ALT (39.77 ± 10.24 U/L), and AST (205.00 ± 12.36 U/L)] significantly attenuated the elevated ALT and AST levels (*P* < 0.05, *P* < 0.01, Figure 1).

**Figure 2:**
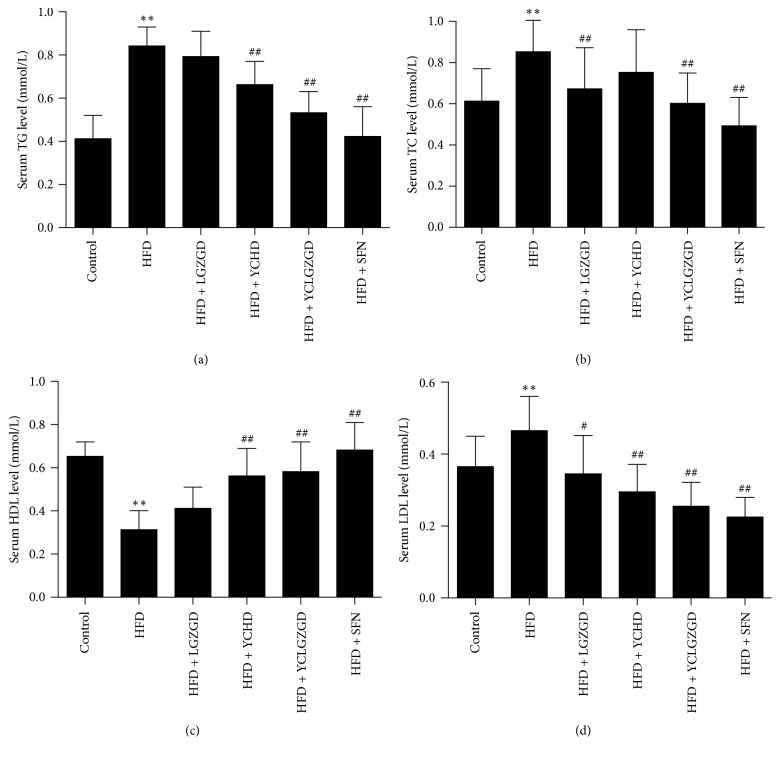
Effect of LGZGD, YCHD, and YCLGZGD on serum levels of TG, TC, HDL, and LDL. Serum levels of (a) TG; (b) TC; (c) HDL; (d) LDL are shown. Data are means ± SD *n* = 10 rats/group; ^*∗∗*^*P* < 0.01 versus control; ^#^*P* < 0.05 and ^##^*P* < 0.01 versus HFD.

TG levels decreased in YCHD [0.67 ± 0.10 mmol/L] and YCLGZGD [0.54 ± 0.09 mmol/L] group compared to that in the HFD model group [0.85 ± 0.08 mmol/L, *P* < 0.01]. LGZGD also decreased TG concentration, but statistical significance was not achieved (*P* > 0.05). Furthermore, LGZGD [0.68 ± 0.19 mmol/L], YCLGZGD [0.61 ± 0.14 mmol/L], and SFN [0.50 ± 0.13 mmol/L, *P* < 0.01] decreased TC concentrations compared to that in the HFD model group [0.86 ± 0.18 mmol/L, *P* < 0.01]. However, YCHD did not decrease TC significantly (*P* > 0.05). LGZGD [0.35 ± 0.10 mmol/L], YCHD [0.30 ± 0.07 mmol/L], YCLGZGD [0.26 ± 0.06 mmol/L], and SFN [0.23 ± 0.05 mmol/L] treatments significantly attenuated the elevated LDL level [0.47 ± 0.09 mmol/L in HFD, *P* < 0.05, *P* < 0.01]. HDL-C levels increased in YCHD [0.57 ± 0.12 mmol/L], YCLGZGD [0.59 ± 0.13 mmol/L], and SFN [0.69 ± 0.12 mmol/L] groups compared to that in the HFD model group [0.32 ± 0.08 mmol/L, *P* < 0.01] (Figure 2). However, the effects of LGZGD were not significantly different compared to the HFD group (*P* > 0.05, Figure 2).

### 3.2. LGZGD, YCHD, and YCLGZGD Treatment Alleviated Hepatic Morphological Changes

**Figure 3:**
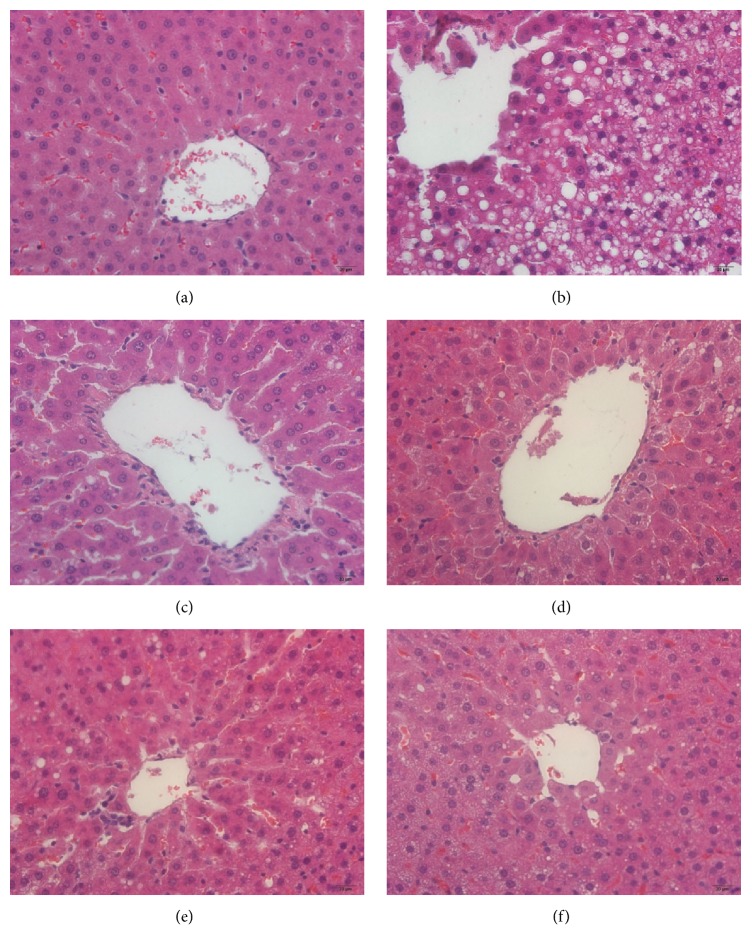
The results of H&E staining, ×200. (a) Control fed rat, (b) HFD model rats, (c) LGZGD treatment rats, (d) YCHD treatment rats, (e) YCLGZGD treatment rats, and (f) SFN treatment rats.

Photomicrographs of the H&E-stained tissue sections showed that the majority of the hepatocytes of HFD-fed rats were distended owing to the presence of fat compared to that reported for the control group (Figures 3(a) and 3(b)), indicating that HFD feeding increased hepatic fat deposits. The H&E-stained sections also displayed steatosis, ballooning degeneration, and infiltration of inflammatory cells in the intercellular substance, which could have caused conspicuous swelling of cells and cytoplasmic vacuolation (Figure 3(b)). Treatment of HFD-fed rats with LGZGD, YCHD, YCLGZGD, and SFN reduced fat deposits in the liver (Figures 3(c), 3(d), 3(e), and 3(f)). Groups treated with LGZGD, YCHD, and YCLGZGD showed lower fat deposits than the HFD group did as shown in Figures 3(c), 3(d), and 3(e).

**Figure 4:**
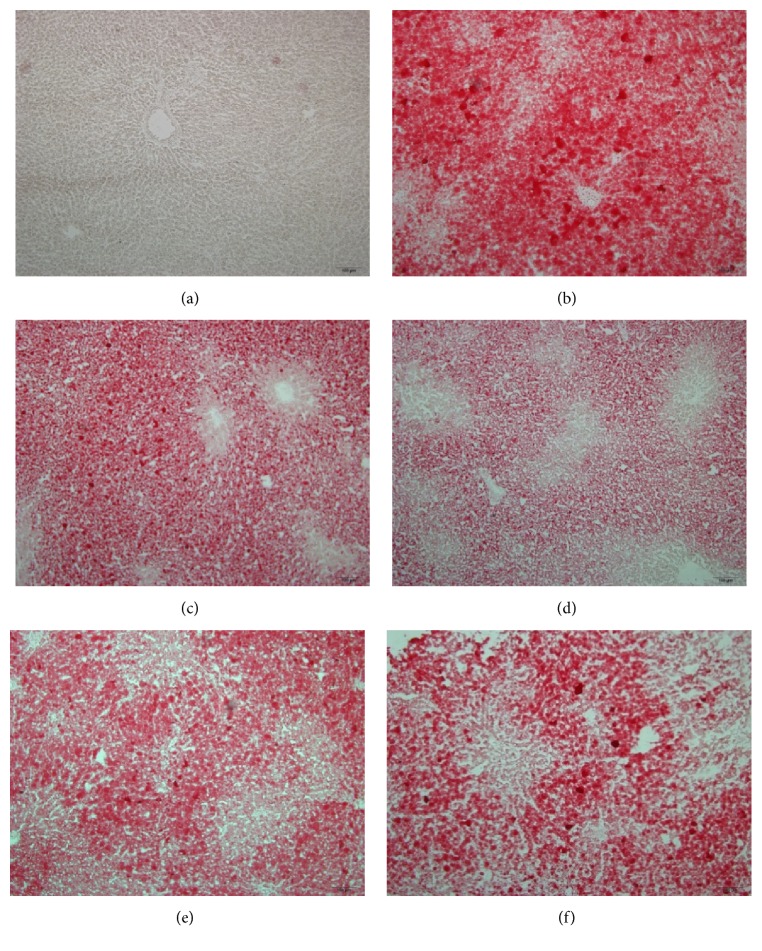
Oil Red O-stained sections, ×100. The red blots show lipid drops in hepatocytes. (a) Control fed rat, (b) HFD model rats, (c) LGZGD treatment rats, (d) YCHD treatment rats, (e) YCLGZGD treatment rats, and (f) SFN treatment rats.

Only few lipid droplets were detected in the ORO-stained frozen liver sections from the control group (Figure 4(a)). Compared to the HFD-fed model rats (Figure 4(b)), treatment with LGZGD, YCHD, YCLGZGD, and SFN remarkably reduced lipid droplets deposited in the hepatocytes (Figures 4(c), 4(d), 4(e), and 4(f)).

### 3.3. LGZGD, YCHD, and YCLGZGD Regulated Hepatic Keap1 Protein and Keap1 mRNA Expression in HFD-Fed Rats

**Figure 5:**
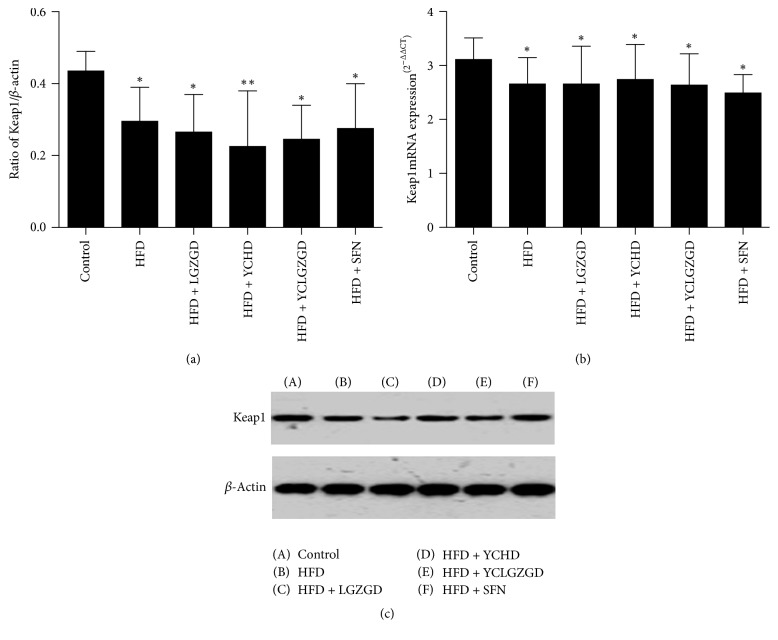
LGZGD, YCHD, and YCLGZGD regulated the hepatic Keap1 protein ((a), (b)) and Keap1 mRNA (c) expression in HFD-fed rats. Control: blank control group; HFD: HFD-fed group; LGZGD: LGZGD treatment group; YCHD: YCHD treatment group; YCLGZGD: YCLGZGD treatment group; and SFN: sulforaphane treatment group. ^*∗∗*^*P* < 0.01 and ^*∗*^*P* < 0.05 versus control group (*n* = 10/group).

The HFD group showed a lower Keap1 expression than the control group did (*P* < 0.05). Similarly, LGZGD, YCHD, YCLGZGD, and SFN treatment groups had a lower Keap1 expression than the control group did (*P* < 0.05). However, there was no significant difference in Keap1 expression between all other groups and HFD group (*P* > 0.05, Figure 5). The expression of Keap1 gene was similar among all six groups.

### 3.4. LGZGD, YCHD, and YCLGZGD Increased Hepatic Nrf2 Protein and Nrf2 mRNA Expression in HFD-Fed Rats

We investigated whether LGZGD, YCHD, and YCLGZGD had a regulatory effect on Nrf2 expression in the liver. Nrf2 levels in the liver significantly increased in the HFD group (*P* < 0.05) compared to that in the control group. LGZGD, YCLGZGD, and SFN treatment showed a sharp increase in Nrf2 expression (*P* < 0.01) compared to that reported for the HFD group.

**Figure 6:**
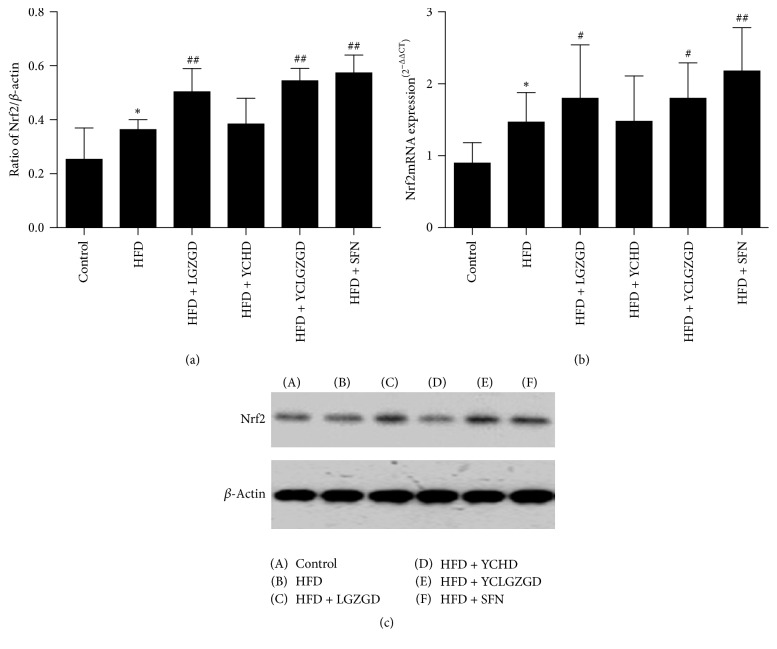
YCLGZGD increased hepatic Nrf2 protein ((a), (b)) and Nrf2 mRNA (c) expression in HFD-fed rats. Control: blank control group; HFD: HFD-fed group; LGZGD: LGZGD treatment group; YCHD: YCHD treatment group; YCLGZGD: YCLGZGD treatment group; and SFN: sulforaphane treatment group. ^*∗*^*P* < 0.05 versus control group; ^#^*P* < 0.05 and ^##^*P* < 0.01 versus HFD group (*n* = 10/group).

Next, Nrf2 gene expression was measured to confirm the effects of YCLGZGD on the liver. As shown in Figure 6, HFD group had a significantly elevated hepatic Nrf2 gene expression compared to that reported for the control group (*P* < 0.05). Treatment with LGZGD, YCLGZGD, and SFN increased Nrf2 gene expression compared to that in the HFD group (^#^*P* < 0.05 and ^##^*P* < 0.01, Figure 6).

### 3.5. LGZGD, YCHD, and YCLGZGD Regulated Hepatic NQO1 Protein and NQO1 mRNA Expression in HFD-Fed Rats

**Figure 7:**
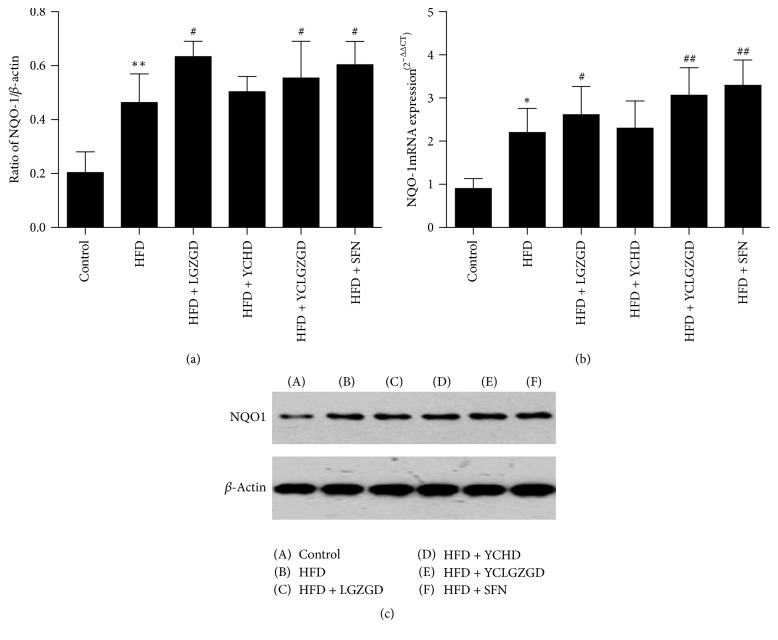
YCLGZGD increased hepatic NQO1 protein ((a), (b)) and NQO1 mRNA expression (c) in HFD-fed rats. Control: blank control group; HFD: HFD-fed group; LGZGD: LGZGD treatment group; YCHD: YCHD treatment group; YCLGZGD: YCLGZGD treatment group; and SFN: sulforaphane treatment group. ^*∗*^*P* < 0.05 versus control group; ^*∗∗*^*P* < 0.01 versus control group; ^#^*P* < 0.05 and ^##^*P* < 0.01 versus HFD group (*n* = 10/group).

The HFD group showed higher NQO1 expression than the control group did (*P* < 0.01). Similarly, LGZGD, YCLGZGD, and SFN groups had a higher NQO1 expression than the HFD group did (*P* < 0.05, Figure 7). The expression of NQO1 gene was similar to the tendency of protein among the four tested groups.

### 3.6. LGZGD, YCHD, and YCLGZGD Treatment Increased Hepatic HO-1 Protein and HO-1 mRNA Expression in HFD-Fed Rats

We investigated whether YCLGZGD had a regulatory effect on HO-1 expression in the liver. HO-1 levels in the liver significantly increased in the HFD group (*P* < 0.05) compared to that in the control group. LGZGD, YCLGZGD, and SFN treatment resulted in a sharp increase in HO-1 expression (*P* < 0.05 and *P* < 0.01 versus the HFD group).

**Figure 8:**
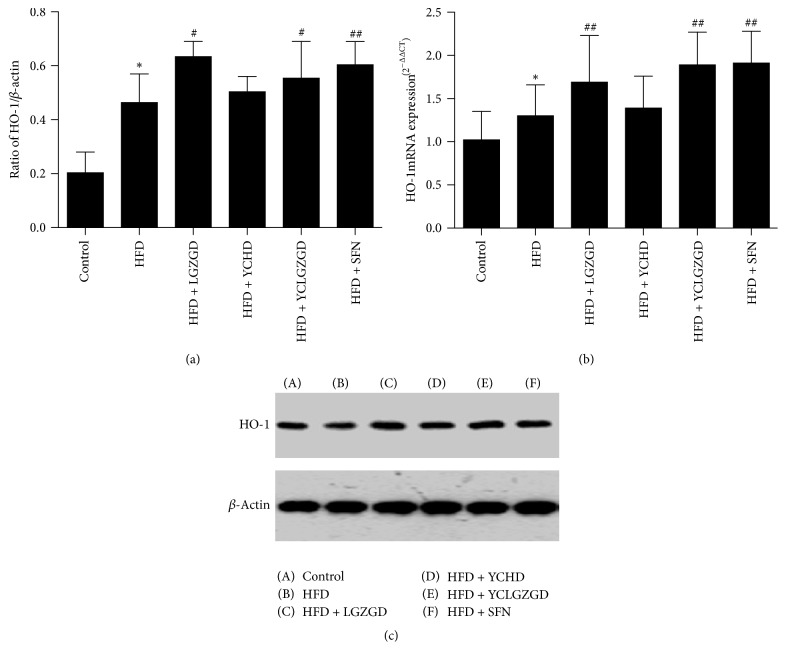
YCLGZGD treatment increased hepatic HO-1 protein ((a), (b)) and HO-1 mRNA (c) expression in HFD-fed rats. Control: blank control group; HFD: HFD-fed; LGZGD: LGZGD treatment group; YCHD: YCHD treatment group; YCLGZGD: YCLGZGD treatment group; and SFN: sulforaphane treatment group. ^*∗*^*P* < 0.05 versus control group; ^#^*P* < 0.05 and ^##^*P* < 0.01 versus HFD group (*n* = 10/group).

Furthermore, HO-1 gene expression was measured to confirm the effects of YCLGZGD on the liver. As shown in Figure 8, HFD group had a significantly higher hepatic Nrf2 gene expression than the control group did (*P* < 0.05). LGZGD, YCLGZGD, and SFN treatment groups showed an increased Nrf2 gene expression compared to that in the HFD group (^##^*P* < 0.01, Figure 8).

## 4. Discussion

NAFLD, a multifactorial disorder caused by various genetic and environmental factors, is considered to be closely associated with hepatic metabolic disorders, resulting in overaccumulation of fatty acids/TGs and cholesterol. The presence of steatosis is closely associated with chronic hepatic inflammation [21], which is mainly caused by oxidative stress and lipid peroxidation (LPO) during the second hit. Free fatty acid oxidation results in production of copious amounts of reactive oxygen species, promoting oxidative stress through several mitochondria-centered pathways. The Nrf2/ARE signaling pathway is involved in this process.

Nrf2 is a key transcription factor that combats cellular oxidative stress. However, whether Nrf2 plays a role in hepatic lipotoxicity is still uncertain. Moreover, the molecular mechanism responsible for the regulation of Nrf2-mediated lipid accumulation remains elusive. Nrf2-null mice exhibited higher lipid accumulation, elevated hepatic fatty acid levels, and oxidative stress after feeding an HFD [22]. Furthermore, the livers of Nrf2-knockout mice fed methionine- and choline-deficient diets exhibited relatively high oxidative stress and inflammation, suggesting that impaired Nrf2 activity might be a risk factor for NAFLD [23–25].

ARE is a nucleotide motif sequence that exists in 5′-upstream promoter region of genes with antioxidative stress. It is widely accepted that Nrf2 is responsible for ARE-dependent gene activation, which can upregulate phase II detoxification and antioxidant enzymes, such as HO-1, NQO1, and superoxide dismutase (SOD).

Keap1 has been shown to interact with the Neh2 (Nrf2-ECH homology domain 2) degron domain of Nrf2 [26]. It is an adaptor subunit of a cullin-3- (CUL3-) based ubiquitin E3 ligase [27]. Under unstressed conditions, Keap1 binds to Nrf2 in the cytoplasm and promotes the ubiquitination and proteasomal degradation of Nrf2. Upon exposure to chemicals (often electrophiles) or reactive oxygen species, the ubiquitin E3 ligase activity of the Keap1–CUL3 complex decreases, and Nrf2 is stabilized. The stabilized Nrf2 accumulates in the nucleus and activates its target genes.

HFD-induced NAFLD animal models have been widely used to identify the pathogenesis and evaluate new treatments [28, 29]. The results of the present study showed that 8 weeks of HFD feeding induced fatty liver disease in SD rats. The rats showed key biochemical features of NAFLD, including elevation of hepatic enzyme levels, hyperlipidemia associated with increased TG accumulation in the liver, histological changes such as steatosis, lobular, and portal inflammation, and hepatocyte injury, for example, ballooning; all of these are characteristics of metabolic syndrome [30]. Furthermore, the histological abnormalities observed in the H&E and ORO-stained liver samples in the HFD-fed rats were consistent with the previous reports [31].

Sulforaphane is an isothiocyanate compound most commonly obtained from cruciferous vegetables [32]. It is produced in plants as a xenobiotic response to predation via vesicular release of the hydrolytic enzyme myrosinase from damaged cells, which converts glucosinolates to isothiocyanates [33]. Over the last two decades, SFN has been extensively characterized for its reported anticancer, antioxidant, and antimicrobial properties [34]. These activities have been mainly attributed to the ability of SFN to modulate the Keap1-Nrf2-ARE signaling pathway.

In previous studies, some researches showed several chemicals which were contained in Chinese herbal medicine such as* Panax notoginseng* [35]*, salviae miltiorrhizae* [36, 37],* Ligusticum wallichii* [38], and* Forsythia suspense* [39, 40] and have Nrf2 activating activities. Few components in YCHD and LGZGD were reported to join in this process except* licorice* [41]. However, the effects of a kind of compound in the formula are not equal to it alone. Formula has the advantage of the whole regulation in different aspects such as improvement in liver enzymes, blood lipid, and symptoms in patients [42].

In the present study, we evaluated the therapeutic efficacy of different treatments in an NASH rat model. Although all three CHM treatments showed therapeutic effects on NASH in rats fed on HFD, the rats in the YCLGZGD group showed more therapeutic improvement, especially in TG and HDL level, than the rats in other two groups did, indicating that LGZGD in combination with YCHD has synergistic effects. We also measured the expression of proteins and genes involved in the Nrf2/ARE signaling pathway. The expression of Nrf2, NQO1, and HO-1 proteins and genes in LGZGD and YCLGZGD treatment groups was similar to that in the SFN group, whereas Keap1 expression was similar between the treatment and HFD groups. This indicates that LGZGD and YCLGZGD regulate the Nrf2/ARE signaling pathway without decreasing Keap1 expression. However, we did not observe obvious effects of YCHD on the proteins and genes in the Nrf2/ARE signaling pathway. YCHD may take advantage of other mechanisms in treating NASH, which need to be explored in the future.

Taken together, the results of the present study showed that YCLGZGD alleviated NAFLD by attenuating oxidative stress and improving lipid regulation, thus providing data to support its clinical use. Because the medicinal herbs present in YCLGZGD have been used in TCM for thousands of years, YCLGZGD is considered safe and tolerable. In conclusion, YCLGZGD can be an optimal approach for NAFLD management given its capacity to regulate oxidative stress, lipid metabolism, inflammatory response, and histological abnormalities via the Nrf2/ARE signaling pathway.
